# Geospatial examination of lithium in drinking water and suicide mortality

**DOI:** 10.1186/1476-072X-11-19

**Published:** 2012-06-13

**Authors:** Marco Helbich, Michael Leitner, Nestor D Kapusta

**Affiliations:** 1 Institute of Geography, University of Heidelberg, Berliner Straße 48, D-69120, Heidelberg, Germany; 2 Department of Geography and Anthropology, Louisiana State University, Baton Rouge, LA, 70803, USA; 3 Department for Psychoanalysis and Psychotherapy, Medical University of Vienna, Währinger Gürtel 18-20, A-1090, Vienna, Austria

**Keywords:** Lithium, Suicide rate, Spatial regression models, Austria

## Abstract

**Background:**

Lithium as a substance occurring naturally in food and drinking water may exert positive effects on mental health. In therapeutic doses, which are more than 100 times higher than natural daily intakes, lithium has been proven to be a mood-stabilizer and suicide preventive. This study examined whether natural lithium content in drinking water is regionally associated with lower suicide rates.

**Methods:**

Previous statistical approaches were challenged by global and local spatial regression models taking spatial autocorrelation as well as non-stationarity into account. A Geographically Weighted Regression model was applied with significant independent variables as indicated by a spatial autoregressive model.

**Results:**

The association between lithium levels in drinking water and suicide mortality can be confirmed by the global spatial regression model. In addition, the local spatial regression model showed that the association was mainly driven by the eastern parts of Austria.

**Conclusions:**

According to old anecdotic reports the results of this study support the hypothesis of positive effects of natural lithium intake on mental health. Both, the new methodological approach and the results relevant for health may open new avenues in the collaboration between Geographic Information Science, medicine, and even criminology, such as exploring the spatial association between violent or impulsive crime and lithium content in drinking water.

## Introduction

One of the first anecdotic evidence of effects of lithium containing water on mental health was mentioned by Cade [1]. Since then, several studies have investigated the relationship between the naturally occurring lithium content in drinking water and suicide mortality [2-5], a relationship which is plausible since lithium is a well recognized mood-stabilizer and suicide preventive in psychiatric treatment nowadays. However, this relationship is also somewhat surprising, as the level of lithium for therapeutic use is multiple times higher than naturally occurring lithium. The first ecological study on the association of lithium in drinking water and suicide rates came from Texas, US. This study was based on aggregated data from 27 counties grouped into low, medium, and high lithium counties and statistically tested by *t*-tests to explore relationships between lithium and crime, suicide, etc. within each of the three groups [2]. Twenty years later, utilizing more comprehensive statistics of weighted ordinary least-squares (OLS) regression, Ohgami et al. [3] confirmed the association using data on lithium in drinking water from 19 municipalities in Oita, a prefecture of Japan. This study sparked interest and was followed by two subsequent studies from Europe. Kabacs et al. [4] examined 47 subdivisions of counties in East England using a Pearson’s correlation coefficient to examine the association, but found results to be not significant. Finally, a nationwide study of all 99 districts of Austria based on the most comprehensive lithium measurement of 6,460 water samples, applied weighted univariate and multivariate OLS, again confirming the suicide preventive potential of natural lithium even after adjustment for confounding factors [5]. However, the main limitation of all these studies is that they do not consider the unique properties of space, having serious consequences concerning the statistical validity of published results (see, e.g. [6-8]).

Essential special properties of geospatial data are spatial autocorrelation and spatial heterogeneity (non-stationarity). The first property implies a spatial association between an attribute value at a particular location and attribute values at other locations close by. The second property describes systematic spatial variation of attribute values across space [6,7]. It is well established that these spatial effects must be taken into account when modeling the spatial relationship in a regression framework [6], such as the association between the amount of lithium in drinking water and mortality ratios or other explanatory variables across space. The consequence of ignoring both spatial effects makes statistical inference invalid and results in misleading conclusions (for a detailed discussion see, e.g. [6-8]).

Therefore, in contrast to all previous research [2-5] this study models both spatial autocorrelation and non-stationarity explicitly by means of global and local spatial statistical models and thus aims to challenge the hypothesis of lithium as a suicide preventive substance in natural distribution.

## Data and methods

The same original data set as in [5] is used in this analysis. In total, 6,460 water samples from drinking water supplies from 99 Austrian districts were analysed for lithium. The average was 65.3 samples per district, with a range from 1 to 312 samples. The lowest measurable threshold lithium level by inductively coupled plasma optical emission spectrometry was 0.0033 mg/l. For the statistical calculations, lithium levels were averaged per district with a mean lithium level of 0.0113 mg/l (SD ± 0.027). Further, data on suicide rates adjusted as standardized mortality ratios (SMR; [9]), proportion of Roman Catholics, population density, and per capita income were obtained from Statistics Austria. Density of psychiatrists, general practitioners, and psychotherapists were obtained from the Austrian Medical Chamber and the Austrian Institute of Health (ÖBIG). Unemployment rates per district were obtained from the Austrian Public Employment Service (AMS) and were averaged for the available years 2005–2008. Further details were described previously in [5].

First, exploratory spatial data analysis is applied to demask spatial patterns which may contradict fundamental model assumptions of the subsequent regression. To start off, spatial autocorrelation and non-stationarity in the variables are explored using the global Moran’s *I* and the local G*-statistic. In this context “global” refers to testing for spatial autocorrelation for the entire study area at once and deriving a single value indicating whether spatial autocorrelation exists and what its strength is.

The Moran’s *I*[10] is a widely used measure of global spatial autocorrelation, which tests whether there are some relationships between location and attribute values. A significant positive statistic indicates that nearby locations of similar attribute values are more spatially clustered than randomly distributed. In contrast, a significant negative statistic shows dissimilar values at nearby locations showing a more dispersed pattern.

This global measure is not capable to explore distinctive local features as well as the non-stationarity of a spatial process. Often, a non-stationary response variable is a first clue for spatially-varying relationships in a multivariate regression model. Therefore, Getis and Ord [11] have introduced the G*-statistic to detect clusters of high or low values by location. High positive values refer to “hot spots” and high negative values to “cold spots”. As such, “hot spots” can be described as areas, where districts with high levels are surrounded by other districts with high levels. In contrast, “cold spots” are clusters with low level districts surrounded by other low level districts. A crucial step in spatial modeling is the choice of an appropriate representation of space. Common choices for such representations are, for instance, contiguity, *k*-nearest neighbors, among others. For a summary see [12].

The classical ordinary least squares (OLS) model is widely used to model the global relationship between a response and one or more explanatory variables. OLS assumes, among other things that residuals are spatially independent. Residual autocorrelation captures unexplained similarities between neighboring districts, which can be a results of omitted variables or a misspecification of the regression model in general [13]. Accounting for spatial effects reduces the magnitude of the prediction error, removes most of the systematic error, and leads to more reliable estimates [8]. In this study the OLS model serves as the reference model.

Global models that account for spatial effects are spatial autoregressive models, introduced by Anselin [6]. They comprise of two special cases, namely the spatial lag and the spatial error model. The spatial lag model extends the standard OLS regression model by including a spatially lagged dependent variable, which can be mostly interpreted as spill-over effects. Ignoring this lagged dependent variable leads the OLS model to be biased and inconsistent [14]. This model will be referred to as the spatial autoregressive (SAR) model in the reminder of this article. The spatial error model addresses the presence of spatial autocorrelation by defining a spatial autoregressive process for the error term and, by doing so, captures unexplained similarities. Not considering this spatial process in the error term yields the OLS estimates to be inefficient, although unbiased [14]. The Lagrange multiplier test statistic (LM) on the estimated OLS residuals helps to decide between these two alternative model specifications.

Global regression models assume a homogeneous behavior of the estimated parameters across space, which has often proven to be unrealistic. A way out is the application of the Geographically Weighted Regression (GWR), a local regression model that explores local spatial variations of the parameters [15]. The GWR models spatial autocorrelation and spatial heterogeneity for subsets of the entire data set. Each subset is established around a regression point with near data points exhibiting a higher influence than more distant data points. The weighting is often based on a bisquare kernel function (e.g. [16]). The setting of an appropriate bandwidth length of the weighting function is crucial. The most common is the adaptive bandwidth, where its length is allowed to vary across space, depending on the density of the data points. In densely populated areas the kernel possesses a shorter bandwidth in contrast to regions with larger inter-point distances, where the bandwidth is longer [15]. A kernel function with an adaptive bandwidth improves the goodness-of-fit, if data points are irregularly distributed across space. Despite the critique that the GWR is more suitable for exploratory analysis (see e.g. [17,18] and references therein), it is a flexible model type to investigate spatially varying relationships, which is the focus of this research. GWR estimations are modeled for the same level of aggregation or spatial units as the original data and can be interpreted in the same way as OLS regression estimates.

## Results

In a step-by-step approach, an exploratory spatial data analysis, a global non-spatial regression model, a global spatial regression model, and finally a local spatial regression model were developed and applied to explore the association that lithium and other factors have on suicide mortality.

### Exploratory (spatial) data analysis

In the analysis to follow, the standardized mortality ratio (SMR) is the dependent variable. The eight independent variables include mean lithium level, population density, per capita income, proportion of Roman Catholics, psychiatrist density, psychotherapist density, general practitioner density, and unemployment rate.

Non-parametric Spearman’s correlations (Table 1) show that suicide mortality is significantly correlated with mean lithium levels per district, population density, per capita income, the proportion of Roman Catholics, as well as the density of psychiatrists and psychotherapist. These six variables are thus potential candidates for influencing suicide mortality in a multivariate regression model. In contrast, the unemployment rate and the general practitioner density did not significantly correlate with the suicide SMR.

**Table 1:** **Spearman correlation*r***_***s***_**between standardized mortality ratios (SMRs) for suicide (2005–2009) and district characteristics**

**District characteristics**	**Suicide SMR**	
	***r*_*s*_**	***p-*val.**
Lithium level, mean (mg/l)	−0.26	0.009 **
Population density (per km^2^)	−0.35	0.000 ***
Per capita income (in 1,000 Euro)	−0.47	0.000 ***
Proportion of Roman Catholics, %	0.37	0.000 ***
Unemployment rate, %	−0.14	0.178
Psychiatrist density (per 10,000)	−0.41	0.000 ***
Psychotherapist density (per 10,000)	−0.49	0.000 ***
General practitioner density (per 10,000)	−0.17	0.099

Signif.: ‘***’ 0.001, ‘**’ 0.01, ‘*’ 0.05.

A significant Moran’s *I* statistic is a first clue that parameter estimates in an OLS regression maybe affected by spatial residual autocorrelation. For this reason, the Moran’s *I* statistic was calculated for the dependent and all eight independent variables included in this study. The neighbourhood relationships for calculating the Moran’s *I* statistic are defined as first order queen contiguity, which is commonly used in applied research (e.g., [16]). Results indicate that both the suicide SMR and three of the eight independent variables exhibit significant global positive spatial autocorrelation. All other five independent variables possess a positive spatial autocorrelation that is not significantly different from a spatially random distribution (Table 2).

**Table 2:** **Global Moran’s*I*statistic for variables included in this study**

	**Moran’s *I***	***p*-val.**
Dependent variable:		
Suicide SMR	0.392	0.001 ***
Independent variables:		
Lithium level	0.400	0.001 ***
Population density	0.015	0.269
Per capita income	0.551	0.001 ***
Prop. of Roman Catholics	0.276	0.001 ***
Unemployment rate	0.060	0.171
Psychiatrist density	0.066	0.132
Psychotherapist density	0.090	0.072
General practitioner density	0.041	0.668

Signif.: ‘***’ 0.001, ‘**’ 0.01, ‘*’ 0.05.

**Figure 1:**
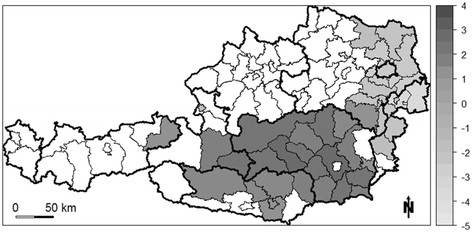
Significant localG*-statistic for suicide SMR (for multiple comparisons*p*-values are adjusted [[19]**, non-shaded districts are not significant).**

The spatial distribution of a variable having significant positive spatial autocorrelation usually shows statistically significant clusters of high (i.e., hot spots) and low values (i.e., cold spots). In Figure 1, significant hot and cold spots of the suicide SMR are depicted using the local G*-statistic. The distribution clearly indicates a large significant cluster of high suicide SMRs in the south (dark gray districts) and a large significant cluster of low suicide SMRs in the eastern portion (light grey districts) of the study area. Districts in white exhibit an insignificant spatial distribution of suicide SMR.

### Global non-spatial regression model

Exploring the relationship between the eight independent variables and suicide SMR starts out with a multivariate OLS regression model. Results show that the eight independent variables explain about 32% of the variance in the suicide SMR. Of those, only lithium levels and the proportion of Roman Catholics are significant predictors (Table 3, left column). Next, a stepwise regression model is estimated with the final model including only those independent variables, for which the regression coefficients are statistically significant by minimizing the Akaike Information Criterion (AIC, see, e.g. [20]). In addition to the explanatory power of a model the AIC also considers model complexity and results in a more parsimonious model [20]. The final stepwise multivariate regression model includes lithium levels, the proportion of Roman Catholics, and the psychiatrist density as the only three independent variables (Table 3, middle column). While the psychiatrist density is not statistically significant, it is still kept in the model by the stepwise algorithm and thus provides explanatory power. Removing this variable from the model would have resulted in an increase of the AIC score from −72 to −59 and a significantly worse performing model. The three independent variabels in the final stepwise model not only explain slightly more of the variation in the suicide SMR (*R*^*2*^_*adj*_ = 0.323 for the stepwise versus *R*^*2*^_*adj*_ = 0.321 for the full model), but also perform significantly better than the full model based on the AIC values (−73 for the stepwise versus −68 for the full model). A more detailed analysis of the residuals from the final stepwise regression model reveals that they are normally distributed (Jarque Bera test (JB) = 1.110; *p* = 0.574) and homoscedastic (studentized Breusch-Pagan test (BP) = 5.133; *p* = 0.162). Because a bivariate correlation analysis of the independent variables showed potential for multicollinearity, Variance Inflation Factors (VIFs) are investigated. Since all VIF scores are below the critical value of 5, as suggested by Fox [21], multicollinearity is rejected. The *F*-test with *p* < 0.001 indicates that the final stepwise regression model is statistically highly significant. Finally, residual independence is tested by the Moran’s *I*. This test shows significant spatial residual autocorrelation (*I* = 0.179; *p* = 0.002), violating the model’s independence assumption. This residual pattern in the stepwise OLS model can be a result of existing spatial effects and can be accounted for by means of a spatial autoregressive model.

**Table 3:** Global regression estimates on standardized mortality ratios (SMRs) for suicide (2005–2009)

	**OLS (full):**			**OLS (stepwise):**			**SAR:**		
	**Coeff.**	***t*-val.**	***p*-val.**	**Coeff.**	***t*-val.**	***p*-val.**	**Coeff.**	***z*-val.**	***p*-val.**
Intercept	0.612	1.908	0.060	0.419	2.491	0.014 *	0.159	0.977	0.329
Lithium level	−7.000	−4.347	0.000 ***	−7.139	−4.847	0.000 ***	−4.844	−3.489	0.000 ***
Population density	0.000	0.565	0.574						
Per capita income	−0.013	−1.039	0.301						
Prop. of Roman Catholics	0.005	2.410	0.018 *	0.006	3.143	0.002 **	0.004	2.573	0.010 **
Unemployment rate	−0.038	−1.597	0.114						
Psychiatrist density	−0.053	−1.110	0.270	−0.041	−1.479	0.142	−0.040	−1.608	0.108
Psychotherapist density	−0.001	−0.116	0.908						
Gen. practitioner density	0.016	1.146	0.255						
ρ							0.448		0.001 ***

Signif.: ‘***’ 0.001, ‘**’ 0.01, ‘*’ 0.05.

### Global spatial regression model

Of the two different types of spatial autoregressive models, the (robust) LM test recommends the use of a spatial lag model (rob. LM: 8.477; *p* = 0.004). Again, variables are selected based on the full model and predictors that do not make a significant contribution on a 0.05 level are omitted stepwise. In the final global spatial lag model, both the lithium level as well as the proportion of Roman Catholics are significant predictors of the overall suicide SMR, the psychiatrist density is nearly significant at α = 0.1 level but its consideration in the model does not increase the AIC (Table 3, right column). Sensitivity analysis with different neigbourhood configurations confirm the robustnes of these results.

A Jarque Bera test (JB = 3.038; *p* = 0.219) shows that the residuals are normally distributed, homoscedastic (BP = 4.303; *p* = 0.231), and no longer significantly different from a spatially random distribution (i.e., they possess no spatial autocorrelation, Moran’s *I*: -0.060; *p* = 0.761). This means that the autoregressive component successfully captures spatial autocorrelation, which is indicated by a significant value of rho (ρ = 0.448; LR: 14.336; *p* < 0.001) and non-significant LM test for residual autocorrelation. In other words, residuals are now in compliance with the assumption of being spatially independent of each other. In addition, the resulting global spatial lag model showed much improvement over the final stepwise OLS model in terms of performance, based on the AIC values (−85 for the global spatial model versus −73 for the stepwise regression model). Overall, the SAR model explains more than 43% (Nagelkerke *R*^*2*^) in the variation of the suicide SMR.

### Local spatial regression model

While the global spatial model is clearly an improvement over the aspatial OLS model, the relationships between the dependent and the independent variables remain stationary (i.e., constant) across the entire study area of Austria. However, multiple studies have indicated that such relationships are in fact non-stationary and thus vary across the study area (e.g., [16]). Local spatial regression models take such non-stationarity into account. Thus, the final step in this analysis involved estimating a GWR that used an adaptive, bisquare kernel function and the significant independent variables indicated by the SAR model.

**Figure 2:**
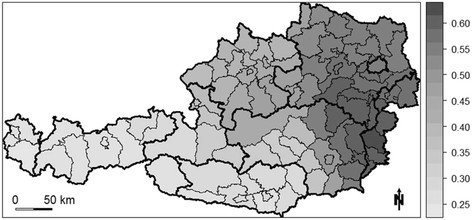
**Local*R***^***2***^**values of the GWR model.**

In the GWR model, lithium level (*p* < 0.05) and proportion of Roman Catholics (*p* < 0.05) show sigificant non-stationarity, meaning that the regression coefficient for both independent variables significantly varies across the study area. However, the Leung et al. [22] test does not reject the null hypotheses of stationarity for the psychiatrist density, which indicates that this explanatory variable seems to have a constant relationship with the suicide SMR across the entire study area. In addition, residuals do not show any spatial autocorrelation. Not only does the GWR model outperform the corresponding global OLS model (*F* = 2.363; *p* < 0.001), it also now explains between about 25% to 60% of the variance in the suicide SMR. As shown in Figure 2, an east–west divide in the explanatory power of the GWR model is apparent, with higher local *R*^*2*^ values in the east and lower values in the western and southern portions of Austria. The highest local *R*^*2*^ values are found in the south-east, leaving approximately 40% of the variation in the suicide SMR unexplained.

**Figure 3:**
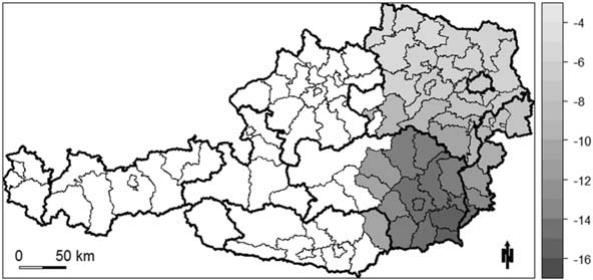
Significant GWR coefficients of the independent variable “lithium level” (white areas indicate regression coefficients that are not significant at α = 0.1).

A statistically significant negative relationship between lithium content in drinking water and suicide mortality can be found in the eastern portion of Austria, including the entire provinces of Burgenland and Vienna, most of Lower Austria and Styria, and eastern portions of Carinthia (Figure 3). In this entire area, the negative relationship is stronger in the southern half and less pronounced in the northern half. For example, a one unit increase in lithium levels in drinking water in one of the southern districts leads to a 16 fold decrease in the suicide SMR. In contrast, the impact of the lithium levels in drinking water on the suicide SMR in one of the northern districts is only ¼ of that in one of the southern districts (Figure 3).

**Figure 4:**
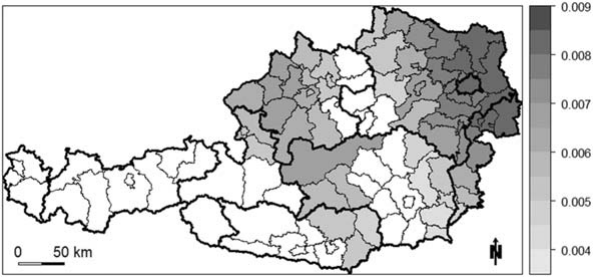
Significant GWR coefficients of the independent variable “proportion of Roman Catholics” (white areas indicate regression coefficients that are not significant at α = 0.1).

**Figure 5:**
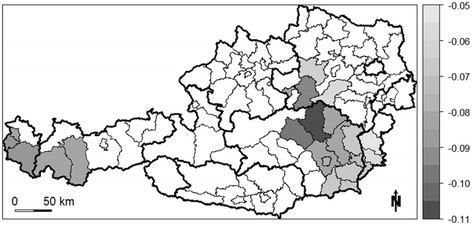
Significant GWR coefficients of the independent variable “psychiatrist density” (white areas indicate regression coefficients that are not significant at α = 0.1)

Significant positive relationships between the proportion of Roman Catholics and suicide SMR can be found mostly for the eastern half of Austria, with the strongest relationships in the north-east (Figure 4). Although, the null hypotheses of spatial stationarity for the psychiatrist density was not rejected (see above), a few districts in the south-eastern and western portions of Austria exhibit a significant negative relationship between the psychiatrist density and the suicide SMR (Figure 5).

## Discussion

Given the increasing possibilities of disease mapping due to a growing availability of digital data in medicine, the experiences of psychiatric epidemiology with geospatial analyses are growing [23]. Up to now, it has been assumed that lithium in drinking water originated from natural sources such as lithium containing rocks [24]. Natural lithium traces in water have already been considered by Cade [1] to have the potential to influence mental health. Since then, the mood-stabilizing effects of lithium became widely recognised by the psychiatric community [25] and in the meanwhile its suicide preventive properties are well documented [26]. As a natural trace element, lithium is washed out by rain from rock and soil and dissolves in ground water and reaches the food-chain via the drinking water. In some geographic regions, its concentrations may reach up to 5.2 mg/l, reflecting a natural daily intake of lithium of up to 10 mg/l [24,27]. Although, such daily doses of lithium are considerably lower than those used therapeutically, it is unknown to which extent intake of natural lithium levels may influence mental health or suicide mortality. Only one randomised placebo-controlled study showed favourable effects of low-dose lithium supplementation (0.4 mg daily) on mood, in a small sample of former drug users [28]. Although, the effects of therapeutic doses of lithium (about 70 mg dissolved lithium per day) are well established, still only little is known about the health effects of natural or low lithium intake as examined in this study.

This study did not consider that lithium prescribed to patients may accumulate via waste water in ground water aquifers due to urinary excretion. There are several reports pointing to the public health problem of antibiotics leading to resistant bacteria in waste water [29,30] as well as pharmaceuticals like carbamazepine, ibuprofen, and biphosphenol A in finished drinking water in Canada [31]. Similarly, acetaminophen, caffeine, carbamazepine, codeine, and other substances are measureable in groundwater from the Los Angeles metropolitan area, when compared to other regions in California [32]. In the United States, also Fluoxetine has been detected in 4.3% of all examined ground water sites [33]. The effects of Fluoxetine and Carbamazepine concentrations found in sediment and water systems were strong enough to affect health and behavior in aquatic invertebrates [34,35]. It is possible that lithium, which is used in psychiatry since more than 60 years, has accumulated in drinking water reservoirs and thus has contributed to the nonrandom distribution of lithium in drinking water across Austrian districts [11]. With this in mind, the found association of lithium in drinking water and suicide mortality could be a function of regional lithium treatment rates rather than an effect of natural lithium occurrence. To answer this question, a further examination of this hypothesis is necessary and spatial regression models are most useful in this regards. Indeed, the authors are already in the process of preparing a project assessing lithium prescription rates at a geographical basis in Austria, which are available and can be purchased.

In particular, local spatial regressions allow to further challenge the hypotheses drawn from linear models. As shown in this study, the associations found between lithium in drinking water vary in strength by region. This underscores the necessity to examine large areas instead of selected regions. For example, the contradicting results in Ohgami at al. [3] and Kabacs et al. [4], which both examined only a random region of each country, a prefecture in Japan and East England, might have been biased by the selection of these regions. Such local spatial regression models are especially informative when multiple risk factors coalesce as causes of a disease. Then, as shown here, strongest risk factors may vary from region to region and such results may be used to inform policy makers and prevention initiatives, which than may be tailored to the local needs in addition to global spatial predictors. While the suicide mortality of the north-east of Austria is best described by the Proportion of Roman Catholics, the south-east is better described by lithium levels. The results suggest that in the south-eastern parts of Austria the density of psychiatrists coincidently exhibits a significant negative relationship with suicide mortality with lithium levels in water, which may be an argument for an influence of local lithium prescriptions by psychiatrists on lithium levels in drinking water.

## Conclusion

The outcomes found here still need replications based on other countries data. Also, replications in other time periods would be informative to substantiate the possible effects of lithium on people’s health. The limitations of the results derived from such ecological models still include the problem of the ecological fallacy in particular, and the Modifiable Areal Unit Problem in general, and do not allow inferring direct causality from such associations but are of generative and explorative value for further basic research. The examined model of suicide mortality is an example for further interesting research questions. May regional patterns of violent crime incidence such as homicides and other violent or impulsive assaults be explained by lithium content in drinking water as previously hypothesized [2].

## Competing interest

The authors do not have financial or non-financial competing interests by publishing this article.

## Authors’ contributions

MH did most of the data analysis, major parts of the writing, and put together all tables and figures. ML had the idea of this research and coordinated it. He also helped with the analysis and the writing. NDK provided all data sets and helped with the analysis and writing, especially with the sections related to lithium and suicide mortality. All authors read and approved the final manuscript.
